# Clinical Potential of Essential Oils: Cytotoxicity, Selectivity Index, and Efficacy for Combating Gram-Positive ESKAPE Pathogens

**DOI:** 10.3390/molecules30193873

**Published:** 2025-09-24

**Authors:** Biruk Bayleyegn Belete, Jerome Ozkan, Parthasarathi Kalaiselvan, Mark Willcox

**Affiliations:** School of Optometry and Vision Science, University of New South Wales, Sydney, NSW 2052, Australia

**Keywords:** essential oils, antibacterial, cytotoxicity, selectivity index, ESKAPE pathogens

## Abstract

(1) Background: Essential oils (EOs) have emerged as promising antibacterial agents due to their broad-spectrum activity and low risk of resistance development. Therefore, this review aimed to assess the effectiveness of EOs against Gram-positive ESKAPE pathogens, and to evaluate their safety and toxicity in mammalian cells. (2) Methods: A comprehensive search was conducted in PubMed, Scopus, and Web of Science. (3) Results: *Heracleum pyrenaicum* exhibited the most potent effect, with a MIC of 0.02–0.04 µg/mL and a selectivity index ranging from 251.3 to 2006.5, indicating high selective toxicity toward bacterial cells over mammalian cells. In contrast, certain species such as *Cannabis* sp. and *Citrus* sp. had selectivity indices of <1, indicating toxicity to mammalian cells. *Ocimum basilicum* showed good efficacy against methicillin-resistant *S. aureus* (MRSA), with a selectivity index of 23.4–34.9, while *Satureja nabateorum* demonstrated potent activity against *E. faecium*, with a selectivity index of 65.6–87.2. (4) Conclusions: EOs from *Heracleum*, *Eucalyptus*, *Cinnamomum*, *Mentha*, *Thymus*, and *Syzygium aromaticum* had good efficacy and high safety margins and show a potential for development for treating Gram-positive ESKAPE pathogen infections. However, EOs with a narrow safety margin (selectivity index < 10) raise concerns and warrant further in vivo and clinical trials to better understand their therapeutic windows and potential adverse effects.

## 1. Introduction

EOs are lipophilic and highly volatile secondary plant metabolites, typically with a molecular weight of approximately 300 Da [1,2]. The term “essential oil” was coined in the 16th century by Paracelsus von Hohenheim. According to the International Organization for Standardization (ISO 9235:1997), an EO is defined as a “product obtained from vegetable raw material, either by distillation with water or steam, or from the epicarp of citrus fruits by a mechanical process, or by dry distillation” [3].

The global demand for EOs has been steadily increasing due to their wide-ranging applications across various commercial industries. Their natural origin, multifunctional properties, and consumer appeal have led to their extensive use in cosmetics, perfumery, food and beverages, spa therapy and relaxation products, sanitation products, agriculture, medicine, and the pharmaceutical sector [4]. The global EOs market size was valued at USD 23.74 billion in 2023 and is projected to reach USD 40.12 billion by 2030, growing at a compound annual growth rate of 7.9% [5]. EOs represent potentially environmentally friendly alternatives in the nutritional, pharmaceutical and healthcare sectors due to their well-documented antimicrobial, anti-inflammatory, anti-proliferative and anti-diabetic biological properties [6].

Over the past 10–15 years, their efficacy and biological activities have been extensively studied [7]. However, their safety and toxicity have been less well studied, although they have been considered as Generally Recognized as Safe (GRAS) products by the Food and Drug Administration (FDA) and Environmental Protection Agency (EPA) in the USA. Several EOs are accepted as food additives by the FDA [8].

The concentration of EOs in commercial products can be 100-to-1000 fold higher compared to their natural concentration in whole plants, in which they typically constitute less than 0.01% [9]. Such high concentrations may pose toxicity risks, particularly when EOs are intensively inhaled [10]. Additionally, their hydrophobic nature can also contribute to cytotoxic effects on mammalian cells [9]. Aldehydes and phenolic compounds in the EOs are known to exhibit high toxicity and can cause adverse events upon direct contact with human tissues such as the eyes, mucous membranes and skin [11]. EOs can also cause skin sensitization, due to oxidation of compounds such as monoterpenes, following exposure to skin [12]. Oxidation during storage may lead to the formation of harmful compounds which have been implicated in adverse effects such as gynecomastia, reportedly associated with lavender and tea tree EOs (TTEO) [13]. Allergic reactions may also occur following inhalation of EOs, although it has also been suggested that such allergic reactions may result from cross-sensitization with other volatile compounds other than EOs themselves [14].

**Figure 1:**
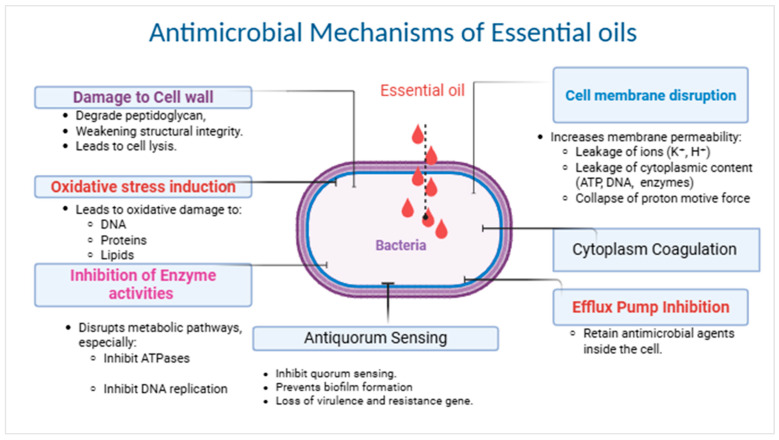
Proposed approaches for how EOs and derivatives combat ESKAPE pathogens [16,18,25,26,27,28,29].

The exact antimicrobial mechanisms of specific EOs against particular bacterial species are still not fully understood [15]. Generally, the antimicrobial properties of EOs are attributed to their lipophilic nature, which enables them to disrupt bacterial cell membranes, leading to increased membrane permeability and loss of intracellular contents. EOs can also penetrate the phospholipid bilayer of the bacterial cell membrane, exerting their inhibitory effects [16,17], as illustrated in Figure 1. Gram-positive bacteria are often more susceptible to EOs than Gram-negative bacteria due to structural differences in their cell envelope. Gram-positive bacteria possess a thick peptidoglycan layer linked with hydrophobic molecules such as proteins and teichoic acid, which may facilitate entry of hydrophobic molecules like EOs [18]. In contrast, Gram-negative bacteria have a more complex cell envelope comprising an outer membrane overlying their peptidoglycan layer, which may make them more resistant to EOs [19]. However, some studies have reported no significant difference in the MIC values of EOs between Gram-positive and Gram-negative bacteria [20,21].

When used as vapors, EOs tend to have increased antimicrobial activity compared to when they are in aqueous solutions. This is due to the need to formulate the EOs for aqueous suspension, such as production of micelles, which may inhibit direct binding of EOs to microbes. Unlike the liquid phase, the vapor phase allows direct binding to microbes and is effective at lower concentrations [22,23,24].

The emergence and rapid spread of antimicrobial resistance among key bacterial pathogens have become a major public health concern worldwide. Among these, the group of pathogens collectively known as ESKAPE (*Enterococcus faecium*, *Staphylococcus aureus*, *Klebsiella pneumoniae*, *Acinetobacter baumannii*, *Pseudomonas aeruginosa*, and *Enterobacter* species) has garnered significant attention due to their ability to escape the effects of conventional antimicrobial therapies [30]. These organisms are responsible for the majority of nosocomial infections and are often associated with high morbidity, mortality, and healthcare costs, particularly in intensive care units [31]. ESKAPE pathogens are characterized by their extensive resistance to multiple antibiotic classes, including last-resort treatments such as carbapenems and vancomycin. They exhibit multidrug resistance (MDR), extensive drug resistance (XDR), and pan-drug resistance (PDR) through various mechanisms, including efflux pumps, enzymatic degradation, target site mutations, and biofilm formation [32,33].

ESKAPE microbes are top priority targets for the development of new antimicrobial strategies by the World Health Organization (WHO) and the Centers for Disease Control and Prevention (CDC) in the USA [34]. According to the 2024 WHO bacterial priority pathogens list (BPPL), Gram-positive ESKAPE pathogens such as vancomycin-resistant enterococci (VRE) and methicillin-resistant *S. aureus* (MRSA) are high priority pathogens [35] for the development of new antimicrobial therapies. In the 2024 WHO BPPL ranking, VRE and MRSA are ranked 8th and 13th on the high priority list [35].

*S. aureus* is a Gram-positive facultative anaerobic bacterium commonly found on the skin, mucosal surfaces and ocular surface of humans [36,37]. While it often exists as a harmless commensal, it can cause a wide range of infections from minor skin infections to life-threatening diseases such as pneumonia, endocarditis, osteomyelitis, sepsis, as well as ocular infections such as keratitis and endophthalmitis which may lead to blindness [38,39,40]. It produces numerous virulence factors, some of which protect the organism from host immune defense systems and antibiotics [41]. The rise in antimicrobial-resistant (AMR) strains has greatly challenged the treatment of *S. aureus* infections, particularly with the emergence of MRSA and multidrug-resistant (MDRSA) strains, which pose a serious threat in both community and healthcare environments [42]. It has been estimated that around 50 million people worldwide carry MRSA strains [43], with very young children, older adults and ill patients being particularly susceptible to infection. The resistance properties of *S. aureus* are due to gene mutations that alter antibiotic targets and cell wall compositions and the acquisition of new genes such as *mecA* (a penicillin binding protein) and *vanA* (a D-Ala-D-Lac ligase) which can make them resistant to β-lactam and vancomycin antibiotics, respectively [44]. 

*E. faecium* is a Gram-positive bacterium that inhabits the gastrointestinal tract as part of the human gut microbiome [45]. However, it can cause a variety of diseases such as bacteraemia, endocarditis, and neonatal meningitis [46]. *E. faecium*, especially VRE, has been identified as the primary MDR *Enterococcus* sp. and has rapidly evolved into a major nosocomial pathogen worldwide. Its remarkable ability to acquire and spread resistance to multiple antibiotics, particularly vancomycin, has contributed to the global emergence of VRE, posing significant challenges for clinical management and hospital infection control [47,48].

Novel and effective approaches are urgently needed to combat these life-threatening diseases caused by these Gram-positive ESKAPE pathogens. Natural products, particularly EOs from medicinal plants, are promising options due to their broad-spectrum antimicrobial activity and the potential to target resistant pathogens through multiple mechanisms of action [49,50,51]. Recent studies show that EOs may provide an effective solution for tackling AMR not only by direct killing pathogens but also by targeting the major determinants of pathogenicity, drug resistance and its spread including cell membrane, drug efflux pumps, quorum sensing, biofilms and R-plasmids [17,52].

The central hypothesis of this review is that certain EOs can be both effective and safe against Gram-positive ESKAPE pathogens in laboratory experiments. Understanding which essential oil extracts from plants are safe in vitro can help identify ones that might be translated into human therapeutics. Therefore, the main objective of this review paper was to evaluate the effectiveness, toxicity and safety of EOs used for treating Gram-positive ESKAPE bacterial infections. To the best of our knowledge, this review is the first to provide a comprehensive analysis that evaluates both the selectivity index (SI) of various EOs and their antimicrobial effectiveness against the main Gram-positive ESKAPE pathogens. By systematically comparing the cytotoxicity and antimicrobial potency of these natural compounds, this review provides critical insights into the potential clinical applicability of EOs as alternative or adjunctive treatments in the fight against MDR infections. By integrating and analyzing data across a range of studies, the review also offers valuable insight into the differential activity of EOs, laying the groundwork for future clinical research. The review also investigates ways of improving the therapeutic efficacy of EOs.

## 2. Methods

Relevant papers were retrieved via electronic searches of PubMed, Medline, ScienceDirect, Scopus, Scientific Electron Library and Cochrane Library, using the terms: “essential oils”, “plant extracts”, “safety”, “cytotoxicity”, “cell lines”, “human”, “animals”, “in vitro”, “in vivo” up to November 2024. These terms were used alone or in combination using Boolean operators (“AND”, “OR”, “NOT”). Only studies that reported both the MIC and the toxicity (which could be the haemolytic, cell-cidal or cell growth-inhibitory concentration) of EOs were included. Furthermore, the studies had to have evaluated the antimicrobial efficacy against strains of *S. aureus* and *Enterococcus* sp. Studies that did not evaluate antimicrobial activity, or that examined the effects of EOs on human physiology (including psychology and inflammatory responses), or their use in agriculture or food, were excluded.

### Selectivity Index of Essential Oils and Its Calculation

Selectivity index, also known as the safety index or therapeutic index, is used to compare the therapeutically effective dose of a substance to its toxic dose [53]. The therapeutic application of EOs is still under investigation and not yet fully understood. Therefore, evaluation of the SI of EOs is important in guiding future investigations, formulation strategies, and safety assessments in both experimental and clinical contexts. Although many studies report the bioactivities of medicinal plants, herbal drugs, and EOs, the lack of a standardized SI limits their scientific credibility and clinical relevance. Challenges in defining the SI of EOs include their complex compositions, lack of standardized dosing, and variability in absorption and metabolism. In the current study, the SI of EOs was calculated as the ratio of the mean cytotoxic concentrations measured using mammalian cell lines, usually reported as the concentration required to kill 50% of cells (CC50), inhibit 50% of cells (IC50), or cause 50% hemolysis of red blood cells (HC50), divided by the mean MIC value of Gram-positive ESKAPE pathogens (usually reported as the concentration required to inhibit the growth of 90% of bacteria) as described previously [54].

$$\mathrm{SI}=\frac{\mathrm{Cytotoxic}\;\mathrm{concentration}\;\mathrm{to}\;\mathrm{mammalian}\;\mathrm{cells}}{\mathrm{MIC}\;\mathrm{value}\;\mathrm{ESKAPE}\;\mathrm{Pathogen}}$$

EOs with a higher SI are theoretically more effective and safer for treating bacterial infections. In other words, the ideal EO would exhibit strong antibacterial activity at very low concentrations while showing cytotoxic effects only at much higher concentrations. This means it may target harmful bacteria without damaging human cells, making it a promising candidate for therapeutic use [55].

The determination of break points of antibacterial agents to define bacterial susceptibility and resistance requires a collaboration of different disciplines such as microbiologists, pharmacologists, infectious disease specialists, data analysis expertise and responsible organizations [56]. Standard MIC break points for various antibiotics have been established by organizations such as Clinical and Laboratory Standards Institute (CLSI), European Committee on Antimicrobial Susceptibility Testing (EUCAST) and partly by Food and Drug Administration (FDA) [57,58,59]. However, for EOs, there are no universally accepted or standardized breakpoints for MIC values to define whether bacteria are susceptible or not, as this area is still being explored.

Based on the data presented, EOs were categorized according to their maximum SI value as follows:

- Very high SI (maximum SI value ≥ 100): These EOs possess a wide safety margin, meaning that their MIC value is significantly lower than cytotoxic dose. Such oils may be relatively safe for use under proper guidance and are less likely to cause adverse effects even when small dosing variations occur.
- High SI (maximum SI value between 10 and 99): EOs within this range are also regarded as relatively safe, though they have a narrower margin of safety compared to those in the very high SI category. Careful dosage control may be important to prevent potential side effects. This has also been confirmed by previous studies, which state that EOs with a SI > 10 are more toxic to various bacteria and fungi with minimal harm to human cells [60].
- Low SI (maximum SI value between 19): EOs with a low SI present a limited safety margin. The MIC dose is closer to the toxic dose, so even minor increases in concentration or exposure duration can result in harmful effects. These oils would require cautious handling and should probably be used under strict supervision, especially in clinical or therapeutic contexts.
- Very low SI (maximum SI value < 1): EOs in this category are considered potentially hazardous, as their toxic dose is equal to or even lower than the MIC value. Such oils pose a significant risk of toxicity, and it is recommended not to be used. If used at all, they should be used with extreme caution, supported by strong clinical evidence and administered by qualified professionals.

These classifications provide insight into the relative safety margins of each EO, with higher SI values indicating a broader safety range between antibacterial MIC value and cell toxic doses. However, it is important to note that the SI may vary depending on the cells used for toxicity, probably due to differences in cellular sensitivity, metabolic activity, and receptor expression, as well as difference in concentration, and exposure time [51].

Most of the cell lines included in this review were derived from cancer cell lines, with a few normal cell lines from both human and animal sources. The detailed information on each cell line is provided including its full name, origin, tissue type, disease association, and cell classification (Supplementary Table S1). This information was obtained from American Type Culture Collection (ATCC), the world’s largest biological resource center (see: ATCC: The Global Bioresource Centre | ATCC), as well as from other reputable sources.

## 3. Results and Discussion

### 3.1. Efficacy and Safety of Essential Oils Against S. aureus

Table 1 presents the MIC, toxicity and calculated SI for different EOs found during the literature search; the EOs are ordered based upon their maximum SI. EOs extracted from the eucalypt *E. cinerea* had a low MIC against *S. aureus* of 0.2 μg/mL [61] with the highest SI (1881.88–3316.3). This suggests the EOs may be safe for use, with minimal toxicity to cells. However, EOs extracted from the eucalypt *E. globulus* had less potent antibacterial activity against *S. aureus*, with reported MIC values ranging from 23 to 330 μg/mL, and had a greater IC50 range (33.2–54,870 μg/mL) [62,63] which resulted in SI ranging from 1.44 to 166.27 (Table 1). The antimicrobial effect of Eucalyptus EOs against *S. aureus* has been shown to be due to a combination of membrane disruption, biofilm inhibition, and multi-target biochemical interference [64]. These effects are primarily driven by 1,8-cineole and other compounds such as α-pinene, limonene, and p-cymene contained with the EOs of *Eucalyptus* [65,66]. The difference in MIC of *E. cinerea* and *E. globulus* could be due to the higher content of 1,8-cineole (eucalyptol), the primary antibacterial component, in *E. cinerea* (79.6%) compared to *E. globulus* (63.1%) [67,68]. Additional variations that may affect MIC and IC50 could result from experimental methodology, including the use of different *S. aureus* strains (ATCC 25923 for *E. cinerea* and PTCC 1337 for *E. globulus*), differing cell incubation times with the EOs (72 h for *E. cinerea* and 4 h for *E. globulus*), as well as the geographical origin of the plant materials (*E. cinerea* from Brazil and *E. cinerea* from Iran). *Eucalyptus* species are native to Australia [69] and have attracted the attention of researchers due to a range of bioactive properties. In addition to their EOs having antimicrobial properties, they also have anti-inflammatory, antioxidant, anti-cancer and immunomodulatory effects [70,71].

The EOs with the lowest MIC (0.01 μg/mL) against *S. aureus* (tested on strain PTCC 1431) was extracted from *Stachys parviflora*. These EOs also had minimal toxicity (high IC50) toward mammalian cells, resulting in very high SIs of 1650–3095 [72]. *Stachys parviflora*, also called *Phlomidoschema parviflorum*, has been traditionally used to treat a variety of ailments such as cough, wound healing, asthma, and epilepsy in addition to its antimicrobial, antioxidant, and antifungal effects [73]. *Cedrus atlantica* (MIC: 0.25 μg/mL; SI: 572.5) [74] and *Foeniculum vulgare Mill*. (MIC: 0.7 μg/mL; SI: 193.5) [75] had good antibacterial activity against *S. aureus*. Other studies [76,77] support the potent antibacterial activity of *Cedrus atlantica* EO, particularly against *S. aureus*. The superior efficacy may be attributed to the EO’s unique chemical composition, which includes bioactive compounds such as himachalene derivatives and α-pinene [78,79], known for their membrane-disruptive properties.

EOs from *Heracleum* species (also known as hogweed) also had good antimicrobial activity against *S. aureus* along with a high SI indicating selective toxicity toward bacterial cells over host cells [80]. *H. pyrenaicum* showed the most potent effect, with a very low MIC of 0.02–0.04 µg/mL and SIs ranging from 251.25 to 2006.5. This was followed by *H. orphanidis* (MIC: 0.02–2.5 µg/mL; SI: 6.62–1247.5), *H. verticillatum* (MIC: 0.14–4.3 µg/mL; SI: 1.37–99.29), *H. pyrenaicum* subsp. *orsinii* (MIC: 0.23–2.59 µg/mL; SI: 2.51–63.7), and *H. ternatum* (MIC: 0.52–1.88 µg/mL; SI: 3.6–34.04) (Table 1). Their antibacterial activity against *S. aureus* has been shown to be due to the presence of several bioactive compounds such as monoterpenes and phenolics which disrupt bacterial membranes, increasing their permeability and causing cytoplasmic leakage, denaturation of proteins and inhibition of enzymes crucial for bacterial survival [80,81]. Additionally, many *Heracleum* species contain furanocoumarins that can enhance antimicrobial efficacy by interfering with DNA replication and generating reactive oxygen species (ROS) and contribute to bacterial cell damage and death [82]. Due to the presence of furanocoumarins, *Heracleum* species have anti-cancer, antioxidant, anti-vitiligo, and immunostimulant effects [82,83].

**Figure 2:**
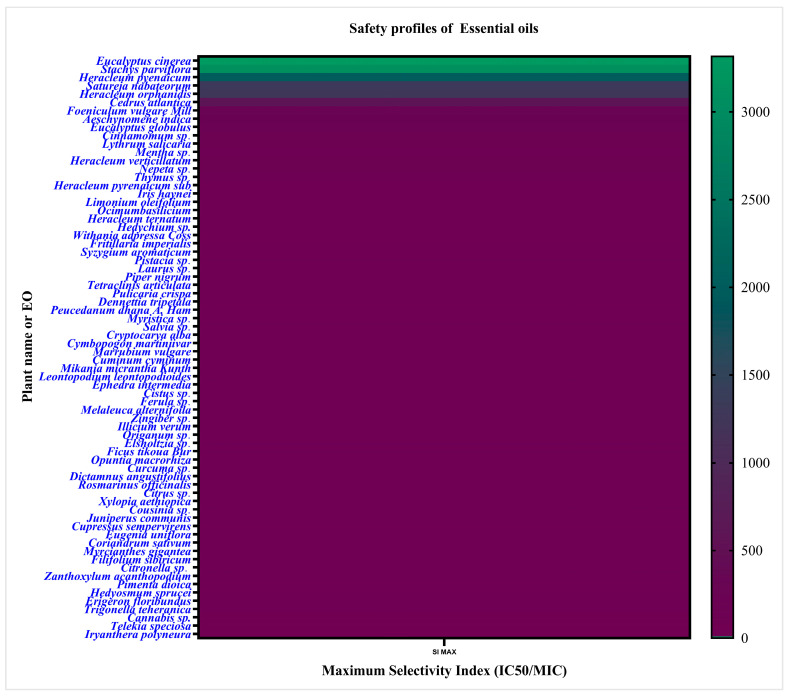
Heatmap illustrating the safety profiles of EOs based on their maximum SI. The heatmap presents the maximum SI values of EOs against mammalian cells in the context of *S. aureus* treatment. A lower SI score reflects higher toxicity to mammalian cells (lower IC50), whereas a higher SI indicates stronger toxicity to bacteria (lower MIC). The color gradient represents the range of maximum SI values irrespective of cell type, with green indicating higher SI values and dark purple color representing lower SI values.

EOs from spice plants such as *Cinnamomum* sp. and *Mentha* sp. are also highly effective against *S. aureus* with high SI values exceeding 100. The EOs *cinnamaldehyde* from *Cinnamomum* sp. and menthone/ol from *Mentha* sp. are the major and relatively safe antimicrobial agents [84,85,86]. Additionally, EOs from *Thymus* (thyme), *Ocimum* (basil), and clove (*Syzygium aromaticum*) have good antimicrobial properties against *S. aureus* with minimal cytotoxic effects on mammalian cells (SI values ranging from 10 to 99; Table 1 and Figure 2).

**Table 1:** Cytotoxicity, MIC and selectivity index of various EOs against *S. aureus* strains (maximum SI from high to low).

Plant Name or EO	Cells Used to Test Toxicity	Toxic (IC50) μg/mL	MIC (μg/mL)	SI MIN	SI MAX	References
*Eucalyptus cinerea*	Jurkat, Hela, Calu-3, HRT-18	391.43–689.8	0.2	1881.9	3316.3 ^a^	[61]
*Stachys parviflora* (*Phlomidoschema parviflorum*)	HCT-116, A2780, B16F10	16.5–30.95	0.01	1650	3095 ^a^	[72]
*Heracleum pyenaicum* (*Heracleum sphondylium* subsp. *pyrenaicum*)	Hela, LS174	10.05–40.13	0.02–0.04	251.3	2006.5 ^a^	[80]
*Satureja nabateorum*	HeLa, HepG2, MCF-7 and COLO-205	82–1090	0.9–12.5	6.6	1282.4 ^a^	[87]
*Heracleum orphanidis*	Hela, LS174	7.5–24.95	0.02–2.5	6.6	1247.5 ^a^	[80]
*Cedrus atlantica*	MCF-7	143.13	0.25	572.5	572.5 ^a^	[74]
*Foeniculum vulgare* Mill	MCF-7	14,060	64	219.7	219.7 ^a^	[75]
*Aeschynomene indica*	MCF-7- HepG2, LO2	40.83–74.09	0.3–0.7	82.1	193.5 ^a^	[88]
*Eucalyptus globulus*	Hela, BHK21, MCF-7, A2780, PC3, DU-145, U-87-MG, C-26	33.2–54,870	23–330	1.44	166.3 ^a^	[63]
*Cinnamomum* sp.	HepG2, HCT-116, MCF-7, MCF10A, HK-2, 786-O, and ACHN	9.1–83,510	7.8–780	8	137.6 ^a^	[81,89]
*Lythrum salicaria*	MCF-7, A2780, PC3, DU-145, U-87-MG and C-26	86	0.63	137.6	137.6 ^a^	[90]
*Mentha* sp.	HaCaT, A 2780, MCF-7, A549, HUVEC	36–382	1.8–40	1.1	108.7 ^a^	[91,92,93]
*Heracleum verticillatum*	HepG2, HCT-116, MCF-7,	5.9–13.9	0.14–4.3	1.4	99.3 ^b^	[81]
*Nepeta* sp.	MCF-7, PC-3, MDA-MB-231, A 2780, LS180, MCF-7, A549, KB and Lymphocyte T	24.9–89.4	0.98–50	0.9	91.2 ^b^	[94,95]
*Thymus* sp.	HeLa, A375, LS174, A549, MRC-5, HepG-2 and PC-3	98.6–485	1.6–5781.3	0.03	86.7 ^b^	[96,97,98,99]
*Heracleum pyrenaicum* subsp. *orsinii*	HeLa, LS174, A549, MRC-5	6.49–13.92	0.2–2.6	2.5	63.7 ^b^	[100]
*Iris haynei*	MCF-7, Hep3B, HepG2, HeLa and Caco-2	757.9–915.47	20.8	36.4	42.1 ^b^	[101]
*Limonium oleifolium*	J774	90.23	2.5	36.1	36.1 ^b^	[102]
*Ocimum basilicium*	HeLa, Hep3B, and MCF-7, and PBMC	53.7–80.35	2.3	23.4	34.9 ^b^	[103,104]
*Heracleum ternatum*	HeLa, LS174, A549 and MRC-5	6.7–17	0.52–1.88	3.6	34.1 ^b^	[105]
*Hedychium* sp.	L929, MRC-5, A549, NCI-H1299, PC-3 and K562	27.6–78,074	312.5–6250	0.04	22.6 ^b^	[106,107,108]
*Withania adpressa* Coss	MCF-12	1000	47	21.3	21.3 ^b^	[109]
*Fritillaria imperialis*	Vero	62.5	3.12–6.5	10	20 ^b^	[110]
*Syzygium aromaticum*	HT31	13,510	780	17.3	17.3 ^b^	[111]
*Pistacia* sp.	MCF-7, HeLa, PC3 and DU-145	37.7–169	12.5–25	1.85	13.5 ^b^	[112,113]
*Laurus* sp.	CaCo-2, MCF-7, MCF10A, B16-F1, and Caco-2	32–324.12	39–64	0.5	6.48 ^c^	[114,115]
*Piper nigrum*	HepG2, HeLa, MCF-7, PC-3 and HEP-2, K562, A549, LS-174, FemX and MRC-5	5.3–56.74	3.9–630	0.04	5.7 ^c^	[116,117]
*Tetraclinis articulata*	RAW 264.7	577.32	125	4.6	4.6 ^c^	[118]
*Pulicaria crispa*	HT-29, MCF-7, Caco-2, Hep-G2	405–1062	236–936	0.4	4.5 ^c^	[119]
*Dennettia tripetala*	RBC	600	150	4.13	4.1 ^c^	[120]
*Peucedanum dhana A. Ham*	Hela, A549, SW480 and 3T3L1	10.24–961.4	250	0.04	3.9 ^c^	[121]
*Myristica* sp.	RAW264.7, H295R and VERO	11.1–440	3.12–12,500	0.01	3.6 ^c^	[122,123]
*Salvia* sp.	HCT-116, L929, A459, HT-29 and MCF-7	32–7000	5000–9500	0.0	3.4 ^c^	[124,125,126]
*Cryptocarya alba*	HK-2, MCF10A	32–64	19	1.7	3.4 ^c^	[115]
*Cymbopogon martiniivar* (*tegi-sar*)	MCF-7, MCF10A	39.23–358	130–500	0.1	2.8 ^c^	[127]
*Marrubium vulgare*	MCF-7	30.1–135.6	50	0.6	2.7 ^c^	[94]
*Cuminum cyminum*	U-87-MG, MCF-7, PC3, DU-145, C-26 and A2780	22.03–41.1	16–19	1.37	2.6 ^c^	[128]
*Mikania micrantha* Kunth	MIAPaCa2, PA1, HeLa and L6	5–82.5	32	0.17	2.6 ^d^	[129]
*Leontopodium leontopodioides*	HCT-116, MCF-7 and 501-MEL	99.2	39	2.5	2.5 ^c^	[130]
*Ephedra intermedia*	HeLa and LnCap	23.22–616.3	250	0.1	2.5 ^c^	[131]
*Cistus* sp.	NIH-3T3, MCF-7, PC-3	14.2–207	70–300	0.1	2.1 ^c^	[132,133]
*Ferula* sp.	HeLa, HepG-2, HT-29, HCT-116, CCRF-CEM and CEM/ADR5000	0.93–252	37.5–2000	0	2 ^c^	[134,135,136,137,138,139]
*Melaleuca alternifoila*	HeLa, K562, A549, LS-174, FemX, MRC-5 and BGM	48.7–265.5	132–310	0.2	2 ^c^	[117,140]
*Zingiber* sp.	HaCaT, A549, PC-3 and K562	10.48–200	78.13–780	0.04	1.4 ^c^	[141,142]
*Illicium verum*	MCF-7	57.3–118.2	100	0.6	1.4 ^c^	[143]
*Origanum* sp.	A549, Vero, Hep2, HT29, MCF-7, NCI-H460, HCT-15, HeLa and HepG2	4–195	12.5–1380	0.06	1.1 ^c^	[144,145,146]
*Elsholtzia* sp.	CaCo2, NIH-3T3, MCF-7, A549 and PC-3	8–828	30–600	0.03	0.9 ^d^	[147,148]
*Ficus tikoua* Bur	NCI-H1299, A549, K562, PC-3 and MRC-5	31.1–130.8	200	0.3	0.8 ^d^	[149]
*Opuntia macrorhiza*	PLP2, MCF-7, HCT15, HeLa and HepG2	206–359	450–1850	0.1	0.8 ^d^	[150]
*Curcuma* sp.	B16 and LNCaP	4.43–429	378–740	0.01	0.6 ^d^	[151]
*Dictamnus angustifolius*	B16	15–57	15–109	0.1	0.5 ^d^	[152]
*Rosmarinus officinalis*	HeLa, A549 and MCF-7	9.9–401.3	1000–2250	0.01	0.4 ^d^	[99,153]
*Citrus* sp.	HeLa, LX-2, K562, A549, LS-174, FemX, MRC-5, HepG2, Caco-2, and HaCaT	25.7–2200	1250–8000	0.02	0.4 ^d^	[117,154]
*Xylopia aethiopica*	RAW 264.7	3.8	16	0.2	0.2 ^d^	[155]
*Cousinia* sp.	A2780, T-47D, A549 and Hep-G2	4.52–32.2	31.3–62.5	0.07	0.1 ^d^	[156]
*Juniperus communis*	HT-29 and HCT116	41–243	2250–6250	0.01	0.1 ^d^	[157]
*Cupressus sempervirens*	AGS	20–289	2500–3000	0.01	0.1 ^d^	[158]
*Eugenia uniflora*	H295R and VERO	101.3–323	3130	0.03	0.1 ^d^	[123]
*Coriandrum sativum*	MCF-7, NCI-H460, HCT-15, HeLa and HepG2	71–140	690–1380	0.05	0.1 ^d^	[146]
*Myrcianthes gigantea*	H295R and VERO	316,6	3130	0.1	0.1 ^d^	[123]
*Filifolium sibiricum*	MCF-7, HepG-2, SKOV-3 and BGC-823	270–780	5200	0.05	0.1 ^d^	[159]
*Citronella* sp.	A431	41.2	500	0.08	0.08 ^d^	[160]
*Zanthoxylum acanthopodium*	SK-LU-1, MCF-7, and HepG-2	16.02–35.5	512	0.03	0.07 ^d^	[161]
*Pimenta dioica*	THP-1	29.6	500	0.06	0.06 ^d^	[162]
*Hedyosmum sprucei*	A549 and MCF-7	42.5–50.9	1000	0.04	0.05 ^d^	[108]
*Erigeron floribundus*	A375, MDA–MB 231 and HCT116	15.9–26.5	512–2048	0.01	0.04 ^d^	[163]
*Trigonella teheranica*	MDA-MB-231, MRC5 and HT-2	3.18–7.82	500	0.01	0.02 ^d^	[164]
*Cannabis* sp.	Caco-2, MCF-7 and MDA-MB-468	22.3–83.2	8000	0	0 ^d^	[165]
*Telekia speciosa*	C32, A375 and HaCaT	7.2	7800	0	0 ^d^	[166]
*Iryanthera polyneura*	MCF-7 and PC-3	6.5–9.8	6000	0	0 ^d^	[167]

^a^ = Very high SI (maximum SI ≥ 100), ^b^ = High SI (maximum SI between 10–99), ^c^ = Low SI (maximum SI between 1–9), ^d^ = Very low SI (maximum SI < 1). See the full name of cells in Supplementary Document.

TTEO is obtained from *M. alternifolia* and is widely recognized for its broad-spectrum antimicrobial activity, with antibacterial, antifungal, antiviral and antiprotozoal activities [168,169]. These have been attributed to the presence of terpinen-4-ol and 1,8- cineole, as major components of the EO, along with α-terpinene, γ-terpinene, terpinolene [169], which disrupt microbial membranes and interfere with vital metabolic pathways. Despite its effective antimicrobial potentials, evidence from two in vitro studies [117,140] indicated that TTEO has low SIs (0.2 and 2) when tested against mammalian cells (Table 1). However, beyond in vitro findings, numerous clinical trials have been conducted. One study reported that TTEO is generally well tolerated, with only minor side effects observed at 25% concentration [170], which, when considering a 25% solution is approximately a thousand times greater than its highest IC50 in Table 1, suggests that in vitro toxicity measurements over-estimate in vivo toxicity. Another clinical trial showed that topical application of 10% TTEO was effective in eradicating MRSA from colonized wounds without any adverse effects or allergic reactions among 32 total participants [171]. There have also been no reports of adverse effects among 60 participants following a 24 h inhalation of TTEO mixed with other EOs over a one-month period [172]. In clinical settings, TTEO has demonstrated considerable promise in the management of various health conditions due to its antimicrobial, anti-inflammatory, and antiseptic properties. It is particularly effective in the treatment of acne, where it has the ability to reduce inflammation and inhibit the growth of *Propionibacterium acnes* (now renamed *Cutibacterium acnes*) [173]. Its efficacy has also been well documented against other superficial diseases including oral candidiasis, tinea, onychomycosis [174]. TTEO is used in the management of wound infections, dermatological conditions and respiratory tract infections such as sinusitis, sore throat, bronchitis, and nasal congestion via inhalation or in vapor therapies [175,176] as well as different chronic ocular inflammatory conditions such as blepharitis and meibomian gland dysfunction [177]. So, while in- vitro toxicity testing can highlight potential risks in clinical settings, it does not necessarily predict whether adverse effects will occur. The main reasons why in vitro assays tend to overestimate toxicity include the absence of whole-organ metabolism, clearance mechanisms, protective barriers, and immune or repair responses in cultured cells [178,179]. In addition, the frequent use of immortalized, cancer-derived, or non-human cell lines can introduce further bias, as these models differ in sensitivity and pathway expression from normal human tissues [180]. Carefully monitored randomized controlled trials are better to validate in vitro findings and ensure safety.

As shown in the heatmap below, several EOs, such as *Eucalyptus* sp. and others with SI > 10 showed strong antibacterial activity with low mammalian toxicity, suggesting potential as safe alternatives or adjuncts to antibiotics. In contrast, oils like *Cannabis* sp. (SI < 10) exhibited antibacterial effects only near cytotoxic levels, indicating a narrow therapeutic window (Figure 2).

### 3.2. Efficacy and Safety of Essential Oils Against MRSA

Whilst *S. aureus* may colonize approximately 30% of individuals, MRSA isolates colonize only about 1% of people [181]. However, due to the resistance of MRSA to beta-lactam antibiotics as well as to other classes such as macrolides, aminoglycosides, glycopeptides, oxazolidinone and lipopeptides [182] and possession of unique pathogenicity traits [183], infection with MRSA is often more difficult to treat.

EOs from TTEO [184], oregano [185], thyme [186], cinnamon [187], frankincense [188] and eucalyptus [189] have potent in vitro activity against MRSA strains. However, only a limited number of investigations have evaluated their cytotoxic effects on human cells in parallel with their antimicrobial properties. Understanding the balance between efficacy and toxicity is crucial for advancing these natural compounds toward clinical application, especially in topical or inhalation-based therapies for MRSA related infections.

As described in Table 2, EOs from *Ocimum basilicum* have relatively low MIC (2.3 μg/mL) against MRSA, with low toxicity to human cells (giving SI of 23.4–34.9). *Ocimum basilicum*, also called sweet basil, is one of the most common culinary and medicinal herbs, widely cultivated in many parts of the world including to Asia, Africa and America [190]. It is rich in bioactive compounds, mainly terpenic compounds, and has not only antibacterial but also antioxidant and antifungal potential [191,192]. Other studies have confirmed that EOs extracted from *Ocimum basilicum* are effective against MRSA [193,194], but their safety and cytotoxicity were not reported.

In contrast, EOs from *Citrus* sp., have limited efficacy against MRSA, with MIC values as high as 25,000 μg/mL. Indeed, EOs derived from *Citrus* plant species generally have low effectiveness against both MRSA and non-MRSA *S. aureus* strains [117,154,195], but may be more active against non-MRSA *S. aureus* [196]. Their application may also possess safety concerns, as evidenced by their low SI (0.02; Table 2 and Figure 2), suggesting that the concentration required for bacterial activity may also be toxic to human cells [195]. The EOs from *Iris haynei* are also more effective against non-MRSA *S. aureus* (MIC: 20.8 µg/mL, SI: 36.4) than MRSA (MIC: 50 µg/mL, SI: 15.2) [101].

### 3.3. Efficacy and Safety of Essential Oils Against E. faecium

*E. faecium* rather than *E. faecalis* has been included in this review because it is more commonly associated with MDR infections in healthcare environments and accounts for up to 90% of human enterococcal infections [197]. However, unlike *S. aureus*, only a limited number of studies have investigated the antibacterial activity of EOs and their toxicity against *E. faecium*.

EOs extracted from *Satureja* sp. had the strongest antibacterial activity against *E. faecium,* with low MIC values (1.25 µg/mL) and relatively high safety margins (SIs ranging from 65.6 to 872) [198]. For these EOs, *E. faecium* had a lower MIC of 1.25 µg/mL compared to *S. aureus*, which has an MIC of 6.6 µg/mL. In contrast, EOs from *Eugenia uniflora* [123], and *Myrcia oblongata* [199] gave higher MIC values (3130 μg/mL and 25,000 μg/mL, respectively) suggesting they are very poorly active against *E. faecium*. The toxic concentrations of these EOs are close to their antimicrobial concentration, resulting in poor SI (Table 2).

**Table 2:** Cytotoxicity, selectivity index, and MIC of various EOs against MRSA and *E. faecium* (maximum SI from higher to lower).

Pathogens	Plant Name or EO	Cells Used to Test Toxicity	Toxic Concentration (IC50) μg/mL	MIC (μg/mL)	SI Min	SI Max	References
MRSA	*Ocimum basilicium*	HeLa, MCF-7, Hep3B	53.7–80.4	2.3	23.4	34.9 ^b^	[103]
	*Iris haynei*	MCF-7, Hep3B, HepG2, HeLa, Caco-2	757.9–915.47	50	15.2	18.3 ^b^	[101]
	*Laurus nobilis*	CaCo-2, MCF-7, B16F1	99.1–324.1	50	2.6	6.5 ^c^	[114]
	*Illicium verum*	HeLa, 3T3, LX-2, MCF-7	57.3–131.7	100	0.6	1.3 ^c^	[143]
	*Citrus* sp.	HeLa, HepG2, LX-2, Caco-2	338–534	25,000	0.01	0.02 ^d^	[195]
*E. faecium*	*Satureja nabateorum*	HeLa, HepG2, MCF-7 and COLO-205	82–1090	1.25	65.6	872 ^a^	[87]
	*Dennettia tripetala*	RBC	620	100	6.2 ^c^	6.2 ^c^	[120]
	*Myrciaria* sp.	H295R	70.1–414.7	780	0.09	0.5 ^d^	[123]
	*Stachys viticina* Boiss	HeLa, Colo-205	250	1600.00	0.16 ^d^	0.16 ^d^	[101]
	*Eugenia uniflora*	H295R, VERO	101.4–323	3130	0.03 ^d^	0.1 ^d^	[123]
	*Abies concolor*	HMEC-1, CRL-1474	0.11–1.38	26	0	0.05 ^d^	[199]
	*Myrcia oblongata*	H295R, VERO, CRL-1474	119.3–440.7	25,000	0	0.02 ^d^	[123,199]

^a^ = Very high SI (maximum SI ≥ 100), ^b^ = High SI (maximum SI between 10–99), ^c^ = Low SI (maximum SI between 1–9), ^d^ = Very low SI (maximum SI < 1). See the full name of cells in Supplementary Document.

### 3.4. Improving Therapeutic Efficacy and Safety of Essential Oils by Nanoencapsulation

The in vitro safety profiles of EOs can be enhanced through nanoencapsulation or nanoemulsion techniques [200,201]. This can address key limitations of EOs such as hydrophobicity, chemical instability, and volatility by improving their handling, dispersibility, stability, and overall bioactivity of these active agents [202]. Various nanoencapsulation systems such as nanoemulsions, solid lipid nanoparticles, nanofibers, liposomes and other have been developed to effectively deliver active compounds [203,204].

Whilst EOs extracted from Eucalyptus are effective antibacterials as well as safe to mammalian cells, their safety profile can be further enhanced through encapsulation in chitosan nanoparticles [205] and liposomal lecithin formulations [206], reducing their toxicity against fibroblast cells. Also, *C. sativa* (hemp) EOs can be toxic to different cell lines (Table 1), but this is reduced after encapsulation of the EOs into nanoemulsions [207]. Encapsulating *Cinnamomum* EO in chitosan nanoparticles reduced cytotoxicity by four-fold compared to the non-encapsulated oil against macrophage cell line RAW 264.7, highlighting their potential for targeted delivery with minimal impact on healthy cells [208]. However, factors such as chitosan concentration, molecular weight, structure, particle size, and zeta potential significantly influence their cytotoxicity [209,210]. A study on the cytotoxicity of free basil oil and its nanoemulsion formulation (composed of basil oil, sorbitan monooleate, Polysorbate 80, and ultrapure water) found that the nanoemulsion did not compromise the viability of healthy human peripheral blood mononuclear cells [104]. Moreover, oregano oil encapsulated in chitosan–alginate nanoparticles exhibited significantly lower cytotoxicity compared to free oregano oil when evaluated using an in vitro cytotoxicity assay on HaCaT cells [211].

Interestingly, encapsulation of EOs may not only enhance their safety profile but also significantly boost their antibacterial potential against *S. aureus* and *E. faecalis* [206,212]. For *M. alternifolia*, the MIC against *S. aureus* decreased from 2000 µg/mL in its free form to 34 µg/mL after liposomal encapsulation, representing approximately a 59-fold decrease when encapsulated [213], and another study using chitosan to encapsulate these EOs reported a lowering of MIC by 2-fold [214]. Likewise, liposomal encapsulated *E. globulus* EOs had a 118-fold reduction, lowering the MIC from 2000 µg/mL to 17 µg/mL [213]. These results highlight the superior antibacterial performance of encapsulated EOs, offering more potent antimicrobial action at significantly lower concentrations. Another study showed that EOs from *Cymbopogon winterianus* with limited antibacterial activity when free, had dramatically increased (1250-fold) activity after encapsulation in liposomes (prepared with soy lecithin) against *S. aureus* and a 625- *E. faecalis* [206]. Encapsulation in chitosan can also improve the antibacterial activity of isolated EOs such as thymol (a key component of *Thymus* oil) and eugenol (a major component of clove oil). Prior to encapsulation, the MICs of thymol and eugenol against *Ralstonia solanacearum* were 175 µg/mL and 275 µg/mL, respectively. After encapsulation, these values dropped substantially to 22.5 µg/mL for thymol and 45 µg/mL for eugenol [215]. Nanoemulsions of *Satureja* EOs with Tween 20 or Tween 80 in Hepes buffer exhibited markedly improved antimicrobial activity, showing reduced MIC values against *S. aureus* (800–1600 µg/mL) compared to the non-encapsulated oils (800–3100 µg/mL) [216]. In a study by Franklyne et al. [217], cinnamon and clove oil nanoemulsion with Brij 35, showed 83–166-fold improvement in static MIC against *S. aureus* compared to the bulk oils. Hence, nanoencapsulation enhances the bioactivity of EOs while reducing their toxicity, making an innovative strategy for developing safe and effective antimicrobial treatment.

## 4. Conclusions

This review has highlighted that certain EOs are emerging as safe and effective natural products with strong potential as alternative treatments for addressing global health challenges such as AMR of the major ESKAPE Gram-positive pathogens. Some EOs have high SI values (≥10) suggesting they are relatively safe with significant antimicrobial activity. These properties make them promising candidates for further development as therapeutic agents, either alone or in combination with antibiotics. However, EOs with SI < 10 may pose safety risks, and their application should be directed by qualified healthcare professionals. Also, encapsulation (especially in chitosan nanoparticles) is a key strategy to enhance the safety and effectiveness of EOs.

## 5. Future Directions

Based on the current findings, several key directions are recommended to advance the therapeutic potential of EOs in combating AMR:

1. Comprehensive cytotoxicity studies and clinical trial: Future research should focus on evaluating the cytotoxic effects of promising EOs using normal (non-cancerous) human cell lines. Large-scale longitudinal clinical trials are also essential to confirm the antimicrobial efficacy and safety of EOs, particularly against MDR pathogens such as MRSA and vancomycin-resistant enterococci. This will help validate their safety profiles and determine appropriate therapeutic doses.
2. Standardization of SI reporting: To support clinical translation, standardized methods for calculating and reporting SI values are needed across studies. Establishing SI thresholds will improve the assessment of EO safety and efficacy.
3. Formulation and delivery systems: Encapsulation techniques (e.g., nano- or micro-encapsulation) should be prioritized to enhance EO activity, help control release, reduce toxicity, and improve bioavailability and stability. Developing EO-based formulations suitable for therapeutic use is a promising area for pharmaceutical innovation.
4. Synergistic studies with antibiotics: Investigating EO-antibiotic combinations could reveal synergistic effects, potentially restoring antibiotic efficacy against resistant strains and lowering required doses, thereby minimizing side effects.
5. Mechanistic studies: Future research should explore the precise mechanisms of action of EOs on bacterial cells to better understand how they inhibit bacterial growth or induce cell death. This knowledge is vital for targeted therapy development.

## 6. Strength and Limitation of the Review

One of the major strengths of this review is that it calculated the SI of various EOs, providing valuable insights into their relative safety and efficacy. Another strength is that it classified the EOs based on their SI values, and this is the first comprehensive report to categorize EOs in this manner. This classification not only highlights the oils with the most promising therapeutic profiles but also serves as a practical reference for future research and potential clinical applications. However, as was outlined with TTEO, SI values may not necessarily translate directly into clinical safety. This may be due to the increased sensitivity of cancer cell lines used to determine cytotoxicity. Several studies have shown that cancer cell lines have increased sensitivity to EOs (reviewed in [218]), hence our call for increased use of non-cancerous cell lines in future experiments.

This review has some limitations, including lack of consideration for variations in incubation times between EO exposure to mammalian cells and to bacteria, which can significantly influence the outcomes of both antimicrobial and cytotoxicity assessments. These limitations suggest areas for improvement in consistency between future studies.

## Data Availability

All data are fully available within the manuscript.

## Supplementary Materials

The following supporting information can be downloaded at: https://www.mdpi.com/article/10.3390/molecules30193873/s1, Table S1: Full name of Cells used in cytotoxicity study.

## Abbreviations

The following abbreviations are used in this manuscript:

AMR	Antimicrobial resistance
BPPL	Bacterial Priority Pathogens List
CDC	Centers for Disease Control and Prevention
EO	Essential Oil
ESKAPE	*Enterococcus faecium*, *Staphylococcus aureus*, *Klebsiella pneumoniae*, *Acinetobacter baumannii*, *Pseudomonas aeruginosa*, and *Enterobacter* Species
FDA	Food and Drug Administration
IC50	Inhibit 50% of Cells
ISO	International Organization for Standardization
MDR	Multidrug Resistance
MDRSA	Multidrug-Resistant *Staphylococcus aureus*
MIC	Minimum Inhibitory Concentration
MRSA	Methicillin Resistant *Staphylococcus aureus*
PDR	Pan-Drug Resistance
SI	Selectivity Index
TTEO	Tea Tree Essential Oil
VRE	Vancomycin resistant *Enterococcus*
WHO	World Health Organization
